# High Mortality in Severe Sepsis and Septic Shock Patients with Do-Not-Resuscitate Orders in East Asia

**DOI:** 10.1371/journal.pone.0159501

**Published:** 2016-07-14

**Authors:** Chun-Ta Huang, Yu-Chung Chuang, Yi-Ju Tsai, Wen-Je Ko, Chong-Jen Yu

**Affiliations:** 1 Department of Internal Medicine, National Taiwan University Hospital, Taipei, Taiwan; 2 Department of Traumatology, National Taiwan University Hospital, Taipei, Taiwan; 3 Graduate Institute of Clinical Medicine, College of Medicine, National Taiwan University, Taipei, Taiwan; 4 Graduate Institute of Biomedical and Pharmaceutical Science, College of Medicine, Fu Jen Catholic University, New Taipei City, Taiwan; 5 Department of Surgery, National Taiwan University Hospital, Taipei, Taiwan; Azienda Ospedaliero-Universitaria Careggi, ITALY

## Abstract

**Background:**

Severe sepsis is a potentially deadly illness and always requires intensive care. Do-not-resuscitate (DNR) orders remain a debated issue in critical care and limited data exist about its impact on care of septic patients, particularly in East Asia. We sought to assess outcome of severe sepsis patients with regard to DNR status in Taiwan.

**Methods:**

A retrospective cohort study was conducted in intensive care units (ICUs) between 2008 and 2010. All severe sepsis patients were included for analysis. Primary outcome was association between DNR orders and ICU mortality. Volume of interventions was used as proxy indicator to indicate aggressiveness of care.

**Results:**

Sixty-seven (9.4%) of 712 patients had DNR orders on ICU admission, and these patients were older and had higher disease severity compared with patients without DNR orders. Notably, DNR patients experienced high ICU mortality (90%). Multivariate analysis revealed that the presence of DNR orders was independently associated with ICU mortality (odds ratio: 6.13; 95% confidence interval: 2.66–14.10). In propensity score-matched cohort, ICU mortality rate (91%) in the DNR group was statistically higher than that (62%) in the non-DNR group (p <0.001). Regarding ICU interventions, arterial and central venous catheterization were more commonly used in DNR patients than in non-DNR patients.

**Conclusions:**

From the Asian perspective, septic patients placed on DNR orders on ICU admission had exceptionally high mortality. In contrast to Western reports, DNR patients received more ICU interventions, reflecting more aggressive approach to dealing with this patient population. The findings in some ways reflect differences between East and West cultures and suggest that DNR status is an important confounder in ICU studies involving severely septic patients.

## Introduction

A do-not-resuscitate (DNR) order is a written advance directive that allows patients to avoid cardiopulmonary resuscitation (CPR) in the event of a cardiopulmonary arrest. The DNR order should be issued cautiously, given that it has dramatic and irreversible consequences.[1] On this account, guidelines for the appropriate use of DNR orders have been published and they have also affirmed that DNR orders are not intended to forgo any other treatment decisions or life-sustaining interventions that may be appropriate.[2, 3] However, there is still confusion about the interpretation of DNR orders around the world. Several studies found that patients with DNR orders are less likely to receive intensive care and life-support measures.[4–6] In reality, the definition of a DNR order does not vary across countries, but the attitude to deal with it does change. It has been recognized that the discrepancies are attributable to the cultural, ethical, historical, political and religious differences.[7–10]

Severe sepsis and septic shock are common and potentially deadly illnesses, affecting millions of people all over the world each year and increasing in incidence.[11] Patients with these critical illnesses always require specialized care in the intensive care units (ICUs). The outcome of septic patients depends on a variety of factors, e.g., the number of acute organ dysfunctions, preexisting comorbidities, need for renal replacement therapy and hemodynamic instability.[12, 13] Undoubtedly, timely and appropriate therapeutic measures also play a pivotal role in determining sepsis outcome.[11]

As we described above, the presence of a DNR order may influence physicians’ decision about whether to order certain treatment modalities not related to CPR. Therefore, it is possible that severely septic patients with DNR orders would have a different outcome as compared to those without DNR orders. However, little is known to date about the impact of the DNR status on the clinical features, care modalities, and outcome of this specific population in the critically ill setting, particularly in East Asia.[14, 15] In this regard, the present study aimed to investigate the differences in the ICU mortality and interventions and procedures between septic patients with and without a DNR order in an Asian country.

## Methods

### Patients

This retrospective observational cohort study was conducted in a medical center in Taiwan. Between January 2008 and December 2010, patients aged 20 years or older and admitted to the ICUs with severe sepsis and septic shock were included in the study.[11] This study was performed in accordance with the ethical standards laid down in the 1964 Declaration of Helsinki and its later amendments. Ethical approval was obtained from the Research Ethics Committee of the National Taiwan University Hospital and the requirement of informed consent was waived.

### Baseline characteristics

Patients were classified as DNR or non-DNR patients according to the DNR status at the time of ICU admission. Other information retrieved included demographics, comorbidities, admission category, and sources of infection. Comorbidities of interest were malignancy, cerebrovascular disease, chronic kidney disease, heart failure, coronary artery disease, liver cirrhosis, hypertension, and diabetes mellitus.[16, 17] Comorbidity burden was assessed by the Charlson comorbidity index (CCI).[18] The admission was divided into two categories: medical and surgical. A surgical admission was defined as having a surgical intervention in 2 weeks prior to ICU admission.[19] On ICU admission, the Acute Physiology and Chronic Health Evaluation (APACHE) II score and Sequential Organ Failure Assessment (SOFA) score were calculated to evaluate sepsis severity.[20, 21] Within 6 h of ICU admission, the types and doses of resuscitation fluids were recorded.

### Sepsis management

Patient care was left to the discretion of the treating intensivists, who were encouraged to adhere to the Surviving Sepsis Campaign guidelines.[22, 23] A checklist to facilitate guideline adherence was available throughout the study period, which consisted of a timetable for resuscitation and supportive measures and the therapeutic endpoints to be achieved.

### Outcome measures

The primary outcome measure of this study was to assess the impact of the DNR status on ICU mortality of severe sepsis and septic shock patients. Other measures of interest included the administration of inotropes and vasopressors, arterial or central venous catheterization, the application of mechanical ventilation, and the use of hemodialysis.

### Statistical analysis

Categorical variables were compared using the χ² test or Fisher’s exact test, as appropriate, and continuous variables using the Student’s t test. Data were reported as number (%) or mean ± standard deviation according to data distribution. Multivariate logistic regression analysis was used to determine the independent factors associated with ICU mortality. Age, sex, and all variables with a p value of <0.1 in the univariate analysis were included in the multivariate model. We also performed the sensitivity analysis by excluding moribund patients who died within 24 h of ICU admission, in that under such circumstances, a DNR order was likely to be issued in response to the imminent death of the subjects. A p value of <0.05 was deemed statistically significant. All analyses were performed using SPSS version 15.0 (SPSS Inc., Chicago, IL).

Since significant differences in baseline characteristics may exist between DNR and non-DNR patient groups, propensity score matching was applied to balance potential confounding variables for outcome measures between the two groups.[24] In this study, the propensity score was the conditional probability for issuing a DNR order, as a binary dependent variable, under a set of measurements. Age, sex, severity scores, comorbidities, admission category, and sources of infection were entered into the model based on biological knowledge. Matching was conducted on a one-to-one basis. The caliper was set to 0.25 standard deviation of the propensity score. The matching procedures were conducted using the Stata software, version 11 (StataCorp, College Station, TX).

## Results

### Study population

**Fig 1:**
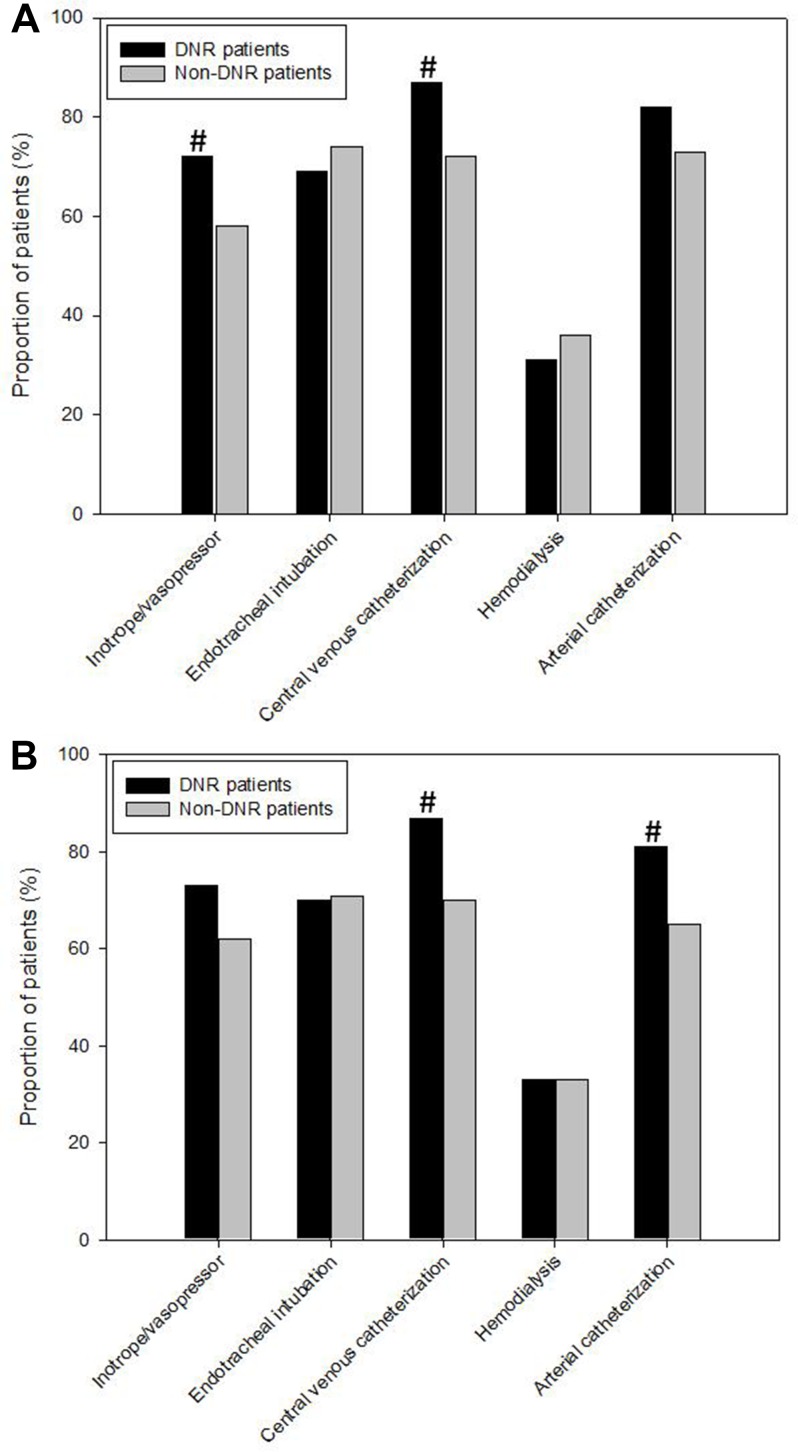
Interventions and procedures during the intensive care unit (ICU) stay according to the do-not-resuscitate (DNR) status on ICU admission. (A) Entire cohort (n = 712); (B) Matched cohort (n = 126). ^#^, significant difference between DNR and non-DNR patients.

During the study period, there were 712 ICU patients admitted for severe sepsis and septic shock. The mean age of the study sample was 63.6 ± 16.0 years. A DNR order was placed on 67 (9.4%) patients on ICU admission. Compared with non-DNR patients, DNR patients were older and more likely to have a medical admission (Table 1). They also had higher disease severity scores and tended to harbor more comorbidities. Inotrope and vasopressor administration and central venous catheterization were more commonly observed in DNR patients (Fig 1A). Of note, these patients experienced extremely high ICU mortality (90%).

**Table 1:** Patient characteristics with regard to the do-not-resuscitate status.

	DNR	Non-DNR	
	n = 67	n = 645	p value
Age, years	70.8±11.9	62.9±16.1	<0.001
Male sex	36 (54)	446 (69)	0.010
APACHE II score	28.7±7.3	23.0±7.1	<0.001
SOFA score	9.8±3.0	7.9±3.5	<0.001
Charlson Comorbidity Index	4.6±3.8	3.9±3.6	0.139
Comorbidities			
Diabetes mellitus	28 (42)	174 (27)	0.010
Hypertension	40 (60)	257 (40)	0.002
Liver cirrhosis	3 (4.5)	39 (6.0)	0.788
Coronary artery disease	10 (15)	79 (12)	0.528
Heart failure	13 (19)	134 (21)	0.792
Chronic kidney disease	12 (18)	103 (16)	0.681
Cerebrovascular disease	2 (3.0)	48 (7.4)	0.216
Malignancy	15 (22)	91 (14)	0.070
Admission category			
Medical	41 (61)	228 (35)	<0.001
Surgical	26 (39)	417 (65)	
Sources of infection			
Pneumonia	25 (37)	247 (38)	0.875
Intra-abdominal infection	18 (27)	225 (35)	0.188
Soft tissue infection	14 (21)	103 (16)	0.300
Others	15 (22)	117 (18)	0.394
Fluid resuscitation			
Crystalloid, ml	2793±1197	2950±1077	0.264
Colloid, ml	414±114	396±87	0.127
ICU mortality	60 (90)	322 (50)	<0.001

APACHE, Acute Physiology and Chronic Health Evaluation; DNR, do-not-resuscitate; ICU, intensive care unit; SOFA, Sequential Organ Failure Assessment.

### Outcome predictors

Patient characteristics with regard to ICU outcome are presented in S1 Table. Multivariate analysis revealed that the presence of a DNR order on ICU admission was significantly associated with ICU mortality (odds ratio: 6.13; 95% confidence interval: 2.66–14.10). The finding was consistent across age, sex, and APACHE II strata (S1 Fig). Unsurprisingly, a higher disease severity score and comorbidity index, and the application of life-sustaining measures, i.e., infusion of inotrope/vasopressor and hemodialysis, were also independent predictors of ICU mortality (Table 2). These results were confirmed in the sensitivity analysis after excluding nine patients (four DNR and five non-DNR) from the primary analysis (S2 Table and S3 Table).

**Table 2:** Multivariate logistic regression analysis to identify independent predictors of intensive care unit mortality.

	Final model		
Variables	Odds ratio	95% confidence interval	p value
APACHE II score, per point	1.05	1.02–1.08	0.001
SOFA score, per point	1.10	1.04–1.16	0.001
CCI, per point	1.05	1.00–1.10	0.038
Sources of infection			
Pneumonia	1.70	1.16–2.47	0.006
Soft tissue infection	1.81	1.11–2.96	0.018
Do-not-resuscitate order	6.13	2.66–14.10	<0.001
Interventions and procedures			
Inotrope/vasopressor	1.78	1.22–2.60	0.003
Hemodialysis	2.58	1.80–3.70	<0.001
Arterial catheterization	2.17	1.40–3.36	0.001

APACHE, Acute Physiology and Chronic Health Evaluation; CCI, Charlson Comorbidity Index; SOFA, Sequential Organ Failure Assessment.

### Propensity score matching

**Fig 2:**
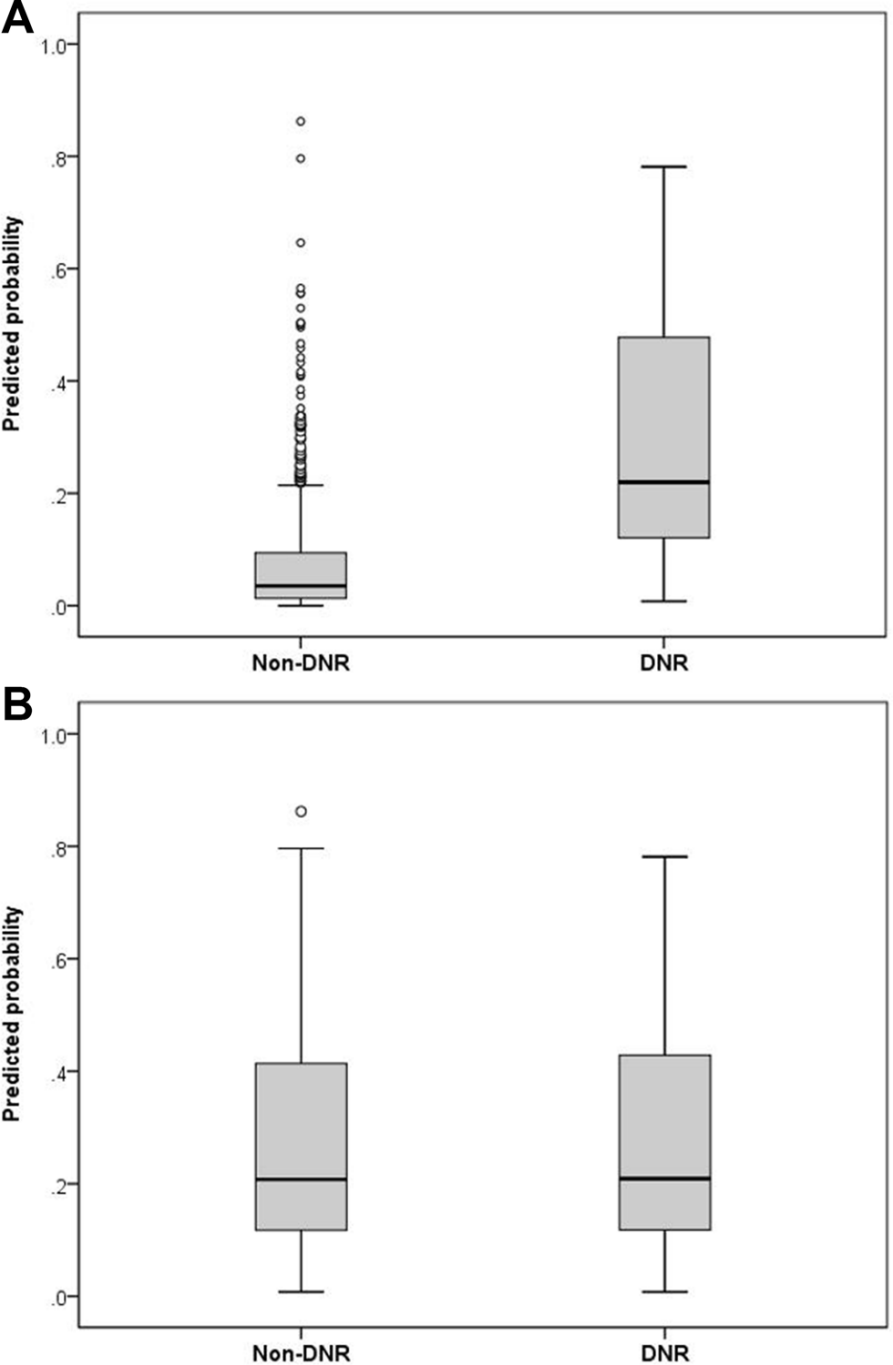
Propensity score matching. Distribution of propensity scores in (A) the do-not-resuscitate (DNR) and non-DNR groups and (B) the matched DNR and non-DNR groups.

Before matching, the propensity scores were markedly different between the two study groups (DNR, 0.28 ± 0.21 vs. non-DNR, 0.07 ± 0.11; p <0.001; Fig 2A). We matched 63 pairs of patients. After matching, the propensity scores were 0.26 ± 0.19 in the DNR group and 0.26 ± 0.20 in the non-DNR group (p = 0.986; Fig 2A). Baseline characteristics, including demographics, severity of critical illnesses, and burden of comorbidities, were similar between DNR and non-DNR patients (Table 3). The ICU mortality rate (91%) in the DNR group was statistically higher than that (62%) in the non-DNR group (p <0.001). Regarding ICU procedures, arterial and central venous catheterization were more commonly used in DNR patients than in non-DNR patients (Fig 1B). The proportions of patients placed on life-support measures, including inotrope/vasopressor infusion, mechanical ventilation, and hemodialysis, were similar between DNR and non-DNR groups.

**Table 3:** Characteristics of propensity score-matched cohort.

	DNR	Non-DNR	
	n = 63	n = 63	p value
Age, years	70.5±12.1	69.2±14.5	0.601
Male sex	32 (51)	30 (48)	0.722
APACHE II score	28.4±7.3	27.3±9.1	0.446
SOFA score	9.9±3.0	10.1±3.9	0.779
Charlson Comorbidity Index	4.7±3.8	5.0±3.9	0.596
Comorbidities			
Diabetes mellitus	26 (41)	27 (43)	0.857
Hypertension	36 (57)	35 (56)	0.857
Liver cirrhosis	3 (4.8)	2 (3.2)	0.999
Coronary artery disease	9 (14)	9 (14)	0.999
Heart failure	13 (21)	15 (24)	0.668
Chronic kidney disease	11 (18)	11 (18)	0.999
Cerebrovascular disease	2 (3.2)	2 (3.2)	0.999
Malignancy	14 (22)	15 (24)	0.832
Admission category			
Medical	37 (59)	42 (67)	0.357
Surgical	26 (41)	21 (33)	
Sources of infection			
Pneumonia	24 (38)	25 (40)	0.855
Intra-abdominal infection	18 (29)	18 (29)	0.999
Soft tissue infection	12 (19)	11 (18)	0.818
Others	13 (21)	10 (16)	0.489
ICU mortality	57 (91)	39 (62)	<0.001

APACHE, Acute Physiology and Chronic Health Evaluation; DNR, do-not-resuscitate; ICU, intensive care unit; SOFA, Sequential Organ Failure Assessment.

## Discussion

This investigation explores the impact of the DNR status on ICU outcome among the critically ill with severe sepsis and septic shock in an East Asian country. We found that the issue of a DNR order on admission was associated with ICU mortality in 90% of severely septic patients. As expected, greater age and severity of illness were associated with DNR orders. In addition, DNR patients were more likely to receive inotrope/vasopressor infusion and central venous catheterization. After adjusting for these confounding variables, a DNR order remained a significant predictor for ICU mortality. In the propensity score-matched cohort, septic patients with DNR orders had a significantly higher ICU mortality rate than did those without DNR orders. DNR patients also underwent more invasive procedures, i.e., arterial and central venous catheterization. The findings in some ways reflect the differences between East and West cultures.

In line with prior studies,[14, 15] a DNR order appears to be an independent risk factor for increased mortality in severe sepsis and septic shock patients. Similar results have been observed in other patient populations, such as stroke, intracerebral hemorrhage, critically ill elderly, and surgery patients.[25–28] Several possibilities exist to explain this phenomenon. First, a DNR order may be interpreted variably to indicate withholding therapeutic measures beyond CPR, e.g., central venous catheters, renal replacement therapy, and intensive care.[4] A recent study on septic shock patients indeed showed that patients with early DNR orders receive fewer interventions and procedures.[15] However, another study and ours did not see such a finding. On the contrary, in our study, septic patients with DNR orders were more likely to undergo invasive procedures. Thus, the association between DNR orders and worse outcome seems not fully attributable to the differences in the aggressiveness of care in the present study.

Second, a DNR order may be placed in response to a rapidly deteriorating disease process and is expected to be closely related to patient outcome. This contention is supported by the observation that the proportion of critically ill patients who are issued DNR orders prior to death is increasing over time.[29, 30] In this regard, we conducted a sensitivity analysis by excluding dying patients and found a result similar to our primary analysis. We therefore suggest that this potential effect did not significantly interfere with our findings.

Third and the most probable possibility is that patients with and without a DNR order differ in their unmeasured or unmeasurable prognostic factors.[27] Despite the fact that we have taken demographics, severity of acute illness, comorbidity burden, and ICU interventions and procedures into consideration, other potentially important confounders, including quality of care, premorbid functional status, patients' will to live, and quality of life, were unavailable for in-depth analysis.[27, 31–33] To understand better which of these covariates is influential on the additional mortality observed in DNR patients, further investigations in this field are required.

The exceptionally high mortality rate observed among septic patients with DNR orders in this study is worthy of attention. Such a high mortality rate has been reported in unselected critically ill patients and in those with acute respiratory distress syndrome, of whom a significant proportion made decisions to withdraw life-support measures.[34, 35] Unlike these studies, we classified only patients with a DNR order on ICU admission into the DNR group, and withdrawal of life-sustaining treatments is rarely an option in the healthcare system in Taiwan.[36] Thus, the possible explanation to the high case fatality is the higher threshold to initiate a DNR order. This speculation is supported by the observation that the rate of DNR orders in our patient cohort (9.4%) is lower than previously reported ones (13.3–19.6%). Moreover, a recent multi-national survey found that Asian intensivists tend to adopt a more aggressive approach to patient care compared to their Western counterparts.[36] As a result, the issue of a DNR order in our study may be an indication that the patient is moving toward the end of life. In the face of such patients with hopeless prognosis and the growing burden of critical illness, how these patients should be treated in the Asian culture remains to be answered.

Multiple patient, institution, and healthcare system-related factors are associated with the application of DNR orders in severely septic patients. Our single-center study found that patient factors associated with having a DNR order included greater patient age, female sex, higher severity scores, and more medical comorbidities. Our findings were in consistent with those from Western studies.[37, 38] Nonetheless, the study design did not allow us to assess the effects of dissimilarities among institutions and healthcare systems on the DNR status. Based on prior studies, we do expect a wide variation in the DNR decisions within and between countries.[8, 10, 36, 38]

The observed overall ICU mortality rate in our study population was 54%, which seemed higher than that (33–43%) reported in other studies.[39–41] Undoubtedly, the outcome of patients with severe sepsis differs substantially across the individual studies. A variety of factors, such as patient age, comorbidities, definition of severe sepsis, types of infection, and disease severity, may contribute to the difference. Indeed, our patient cohort exhibited a higher APACHE II score as compared to other study populations and might, as expected, have a poorer outcome.[40, 41]

Our study carries a number of limitations. First, a single-institution study limited the generalizability of the study results. This, however, is not a concern because varying attitudes about the DNR order are observed around the world and we aimed to share our regional experience from the Asian perspective.[8, 10, 36, 38] Second, certain unmeasured factors may influence the variables under study, but we believe that the large effect size of the association between a DNR order and ICU mortality in the multivariate analysis is hardly false. Finally, given the retrospective nature of the study, intensivists', patients', and families' viewpoints on the DNR orders in septic patients are difficult to be understood from the chart records. These qualitative effects may also have an impact on patient care and outcome. The issue should be specifically tackled in future prospective studies.

In conclusion, in the Asian culture, severe sepsis and septic shock patients who were placed on DNR orders on ICU admission had exceptionally high mortality. A number of patients factors, e.g., age and disease severity, were involved in the determination of a DNR order. In contrast to the Western reports, septic patients with DNR orders in our study received more ICU procedures, reflecting a more aggressive approach to dealing with this patient population in Taiwan. Owing to the growing interest in the end-of-life decisions around the world, our study not only confirms the influential effects of a DNR order on septic patient outcome but also indicates the disparities in care across cultures. We suggest that the DNR status is an important confounder in ICU studies involving severely septic patients and warrants to be taken into account in future investigations.

## Supporting Information

S1 Dataset Spreadsheet containing the data used in this study. (XLSX)

S1 Fig Intensive care unit mortality risk for patients with a do-not-resuscitate order: Stratification analysis by patient characteristics. APACHE, Acute Physiology and Chronic Health Evaluation; CI, confidence interval.
(TIF)

S1 Table Characteristics of the study population with regard to intensive care unit survival. (DOCX)

S2 Table Patient characteristics in the sensitivity analysis after excluding those patients who were dead within 24 hours of admission. (DOCX)

S3 Table Multivariate logistic regression model to identify predictors of intensive care unit mortality in the sensitivity analysis. (DOCX)
