# Supplementary figures and images for: High Mortality in Severe Sepsis and Septic Shock Patients with Do-Not-Resuscitate Orders in East Asia
Source: PLoS One. 2016 Jul 14;11(7):e0159501. doi: 10.1371/journal.pone.0159501 (PMC4944975; doi:10.1371/journal.pone.0159501)

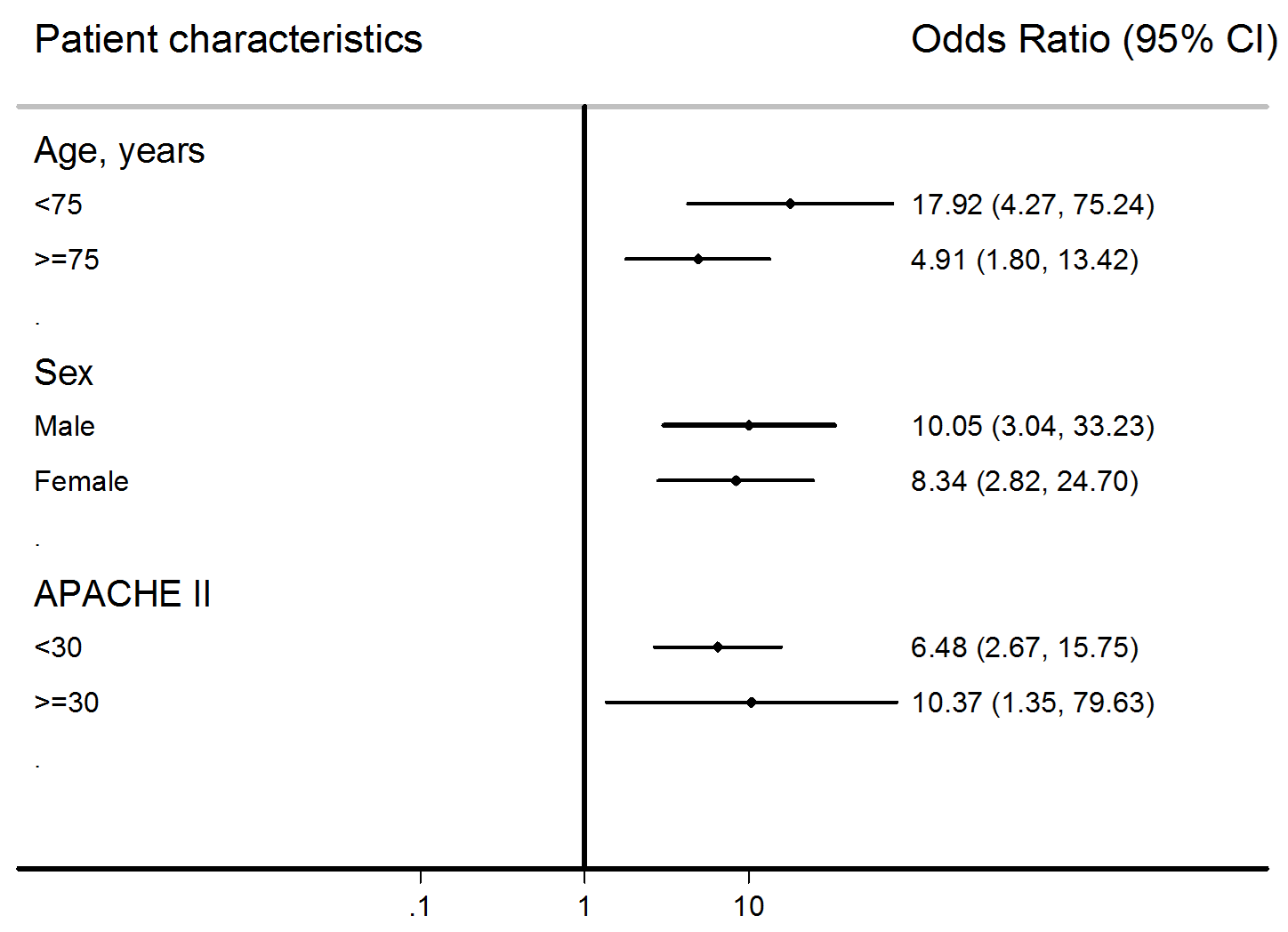

Supplement: S1 Fig — APACHE, Acute Physiology and Chronic Health Evaluation; CI, confidence interval. (TIF) [file pone.0159501.s002.tif]
