# Supplementary material for: High Mortality in Severe Sepsis and Septic Shock Patients with Do-Not-Resuscitate Orders in East Asia
Source: PLoS One. 2016 Jul 14;11(7):e0159501. doi: 10.1371/journal.pone.0159501 (PMC4944975; doi:10.1371/journal.pone.0159501)
Supplement: S1 Table — (DOCX) [file pone.0159501.s003.docx]

S1 Table. Characteristics of the study population with regard to intensive care unit survival

|  | Survivors  n=330 | Non-survivors  n=382 | p value |
| --- | --- | --- | --- |
| Age, years | 63.1±16.3 | 64.0±15.7 | 0.457 |
| Male sex | 216 (66) | 266 (70) | 0.234 |
| APACHE II score | 21.4±6.1 | 25.4±7.7 | <0.001 |
| SOFA score | 7.0±3.0 | 9.0±3.7 | <0.001 |
| Charlson Comorbidity Index | 3.7±3.3 | 4.2±3.8 | 0.048 |
| Comorbidities |  |  |  |
| Diabetes mellitus | 85 (26) | 117 (31) | 0.151 |
| Hypertension | 129 (39) | 168 (44) | 0.187 |
| Liver cirrhosis | 18 (5.5) | 24 (6.3) | 0.640 |
| Coronary artery disease | 33 (10) | 56 (15) | 0.061 |
| Heart failure | 67 (20) | 80 (21) | 0.834 |
| Chronic kidney disease | 53 (16) | 62 (16) | 0.951 |
| Cerebrovascular disease | 25 (7.6) | 25 (6.5) | 0.591 |
| Malignancy | 42 (13) | 64 (17) | 0.132 |
| Admission category |  |  |  |
| Medical | 111 (34) | 158 (41) | 0.034 |
| Surgical | 219 (66) | 224 (59) |  |
| Sources of infection |  |  |  |
| Pneumonia | 115 (35) | 157 (41) | 0.087 |
| Intra-abdominal infection | 124 (38) | 119 (31) | 0.071 |
| Soft tissue infection | 46 (14) | 71 (19) | 0.095 |
| Others | 62 (19) | 70 (18) | 0.874 |
| Do-not-resuscitate order | 7 (2.1) | 60 (16) | <0.001 |
| Fluid resuscitation |  |  |  |
| Crystalloid, ml | 2975±1121 | 2901±1060 | 0.361 |
| Colloid, ml | 392±83 | 403±95 | 0.198 |
| Interventions and procedures |  |  |  |
| Inotrope/vasopressor | 153 (46) | 270 (71) | <0.001 |
| Endotracheal intubation | 241 (73) | 281 (74) | 0.873 |
| Central venous catheterization | 204 (62) | 315 (83) | <0.001 |
| Hemodialysis | 75 (23) | 178 (47) | <0.001 |
| Arterial catheterization | 209 (63) | 319 (84) | <0.001 |

APACHE, Acute Physiology and Chronic Health Evaluation; SOFA, Sequential Organ Failure Assessment.
