# Supplementary material for: High Mortality in Severe Sepsis and Septic Shock Patients with Do-Not-Resuscitate Orders in East Asia
Source: PLoS One. 2016 Jul 14;11(7):e0159501. doi: 10.1371/journal.pone.0159501 (PMC4944975; doi:10.1371/journal.pone.0159501)
Supplement: S2 Table — (DOCX) [file pone.0159501.s004.docx]

S2 Table. Patient characteristics in the sensitivity analysis after excluding those patients who were dead within 24 hours of admission

|  | Survivors  n=330 | Non-survivors  n=373 | p value |
| --- | --- | --- | --- |
| Age, years | 63.1±16.3 | 64.0±15.8 | 0.452 |
| Male sex | 216 (66) | 260 (70) | 0.229 |
| APACHE II score | 21.4±6.1 | 25.2±7.6 | <0.001 |
| SOFA score | 7.0±3.0 | 8.9±3.6 | <0.001 |
| Charlson Comorbidity Index | 3.7±3.3 | 4.2±3.8 | 0.074 |
| Comorbidities |  |  |  |
| Diabetes mellitus | 85 (26) | 113 (30) | 0.182 |
| Hypertension | 129 (39) | 162 (43) | 0.244 |
| Liver cirrhosis | 18 (5.5) | 23 (6.2) | 0.688 |
| Coronary artery disease | 33 (10) | 53 (14) | 0.089 |
| Heart failure | 67 (20) | 77 (21) | 0.911 |
| Chronic kidney disease | 53 (16) | 61 (16) | 0.916 |
| Cerebrovascular disease | 25 (7.6) | 23 (6.2) | 0.460 |
| Malignancy | 42 (13) | 60 (16) | 0.207 |
| Admission category |  |  |  |
| Medical | 111 (34) | 155 (42) | 0.031 |
| Surgical | 219 (66) | 218 (58) |  |
| Sources of infection |  |  |  |
| Pneumonia | 115 (35) | 155 (42) | 0.068 |
| Intra-abdominal infection | 124 (38) | 115 (31) | 0.060 |
| Soft tissue infection | 46 (14) | 69 (19) | 0.103 |
| Others | 62 (19) | 68 (18) | 0.849 |
| Do-not-resuscitate order | 7 (2.1) | 56 (15) | <0.001 |
| Interventions and procedures |  |  |  |
| Inotrope/vasopressor | 153 (46) | 266 (71) | <0.001 |
| Endotracheal intubation | 241 (73) | 278 (75) | 0.651 |
| Central venous catheterization | 204 (62) | 309 (83) | <0.001 |
| Hemodialysis | 75 (23) | 177 (48) | <0.001 |
| Arterial catheterization | 209 (63) | 313 (84) | <0.001 |

APACHE, Acute Physiology and Chronic Health Evaluation; SOFA, Sequential Organ Failure Assessment.
