# Supplementary material for: High Mortality in Severe Sepsis and Septic Shock Patients with Do-Not-Resuscitate Orders in East Asia
Source: PLoS One. 2016 Jul 14;11(7):e0159501. doi: 10.1371/journal.pone.0159501 (PMC4944975; doi:10.1371/journal.pone.0159501)
Supplement: S3 Table — (DOCX) [file pone.0159501.s005.docx]

S3 Table. Multivariate logistic regression model to identify predictors of intensive care unit mortality in the sensitivity analysis

|  | Final model | |  |
| --- | --- | --- | --- |
| Variables | Odds ratio | 95% confidence interval | p value |
| APACHE II score, per point | 1.05 | 1.02-1.08 | 0.001 |
| SOFA score, per point | 1.09 | 1.03-1.15 | 0.004 |
| CCI, per point | 1.05 | 1.00-1.10 | 0.049 |
| Sources of infection |  |  |  |
| Pneumonia | 1.46 | 1.03-2.08 | 0.034 |
| Do-not-resuscitate order | 5.83 | 2.53-13.43 | <0.001 |
| Interventions and procedures |  |  |  |
| Inotrope/vasopressor | 1.78 | 1.22-2.61 | 0.003 |
| Hemodialysis | 2.65 | 1.85-3.79 | <0.001 |
| Arterial catheterization | 2.18 | 1.41-3.38 | <0.001 |

APACHE, Acute Physiology and Chronic Health Evaluation; CCI, Charlson Comorbidity Index; SOFA, Sequential Organ Failure Assessment.
